# Is Maternal Smoking during Early Pregnancy a Risk Factor for All Low Birth Weight Infants?

**DOI:** 10.2188/jea.JE2007415

**Published:** 2008-05-29

**Authors:** Kohta Suzuki, Taichiro Tanaka, Naoki Kondo, Junko Minai, Miri Sato, Zentaro Yamagata

**Affiliations:** 1 Department of Health Sciences, School of Medicine, University of Yamanashi

**Keywords:** Infant, Low Birth Weight, Pregnancy, Risk Factors, Smoking

## Abstract

**Background:**

Low birth weight (LBW) infants do not form a homogeneous group; LBW can be caused by prematurity or poor fetal growth manifesting as small for gestational age (SGA) infants or intrauterine growth retardation. We aimed to clarify the relationship of maternal smoking with both SGA and preterm LBW infants.

**Methods:**

The study population comprised pregnant women who registered at the Koshu City between January 1, 1995, and December 31, 2000, and their children. We performed multivariate analyses using multiple logistic regression models to clarify the relationship of maternal smoking during pregnancy with the SGA outcome and preterm birth in LBW infants.

**Results:**

In this study period, 1,329 pregnant women responded to questionnaires, and infant data were collected from 1,100 mothers (follow-up rate: 82.8%). The number of LBW infants was 81 (7.4%). In this cohort, maternal smoking during early pregnancy was associated with LBW and the SGA outcome. Maternal smoking during early pregnancy was a risk factor for LBW with SGA outcome and for LBW with full-term birth. However, it was not a risk factor for LBW with appropriate weight for gestational age (AGA) and LBW with preterm birth.

**Conclusion:**

These results suggested that LBW with AGA and LBW with preterm birth were associated with other risk factors that were not considered in this study, such as periodontal disease. For the prevention of LBW, not only abstinence from smoking during pregnancy but also other methods such as establishing a clinical setting should be adopted.

## INTRODUCTION

In recent years, Japan has witnessed an increase in the incidence of low birth weight (LBW) infants. In 1975, 5.5% of all infants born weighed less than 2,500 g, while in 2003, this value increased to 10.1%.^1^ Contrastingly, other industrialized countries such as the US, Canada, Sweden, and Norway have witnessed an opposite trend, i.e., a decrease in the prevalence of LBW, during the same period.^2^ LBW is a more important public health problem in Japan than in other industrialized countries.

The cause of LBW infants can be either preterm birth or poor fetal growth manifesting as small for gestational age (SGA) infants or intrauterine growth retardation (IUGR). Moreover, LBW, SGA, and prematurity or a combination of these outcomes are closely related to neonatal and long-term morbidity.^3^^,^^4^ Preterm birth accounts for a large proportion of neonatal deaths.^5^^,^^6^ It is also increasingly recognized that SGA babies have an increased risk of developing chronic diseases in adulthood, such as hypertension, type-2 diabetes, and coronary heart disease.^7^^,^^8^

Maternal smoking during pregnancy is a strong dose-dependent risk factor for LBW.^9^^-^^11^ It also increases the risk of preterm birth^6^^,^^9^ although it appears to affect fetal growth more than gestational duration.^5^

The relationship between maternal smoking during pregnancy and each of these pregnancy outcomes, namely, LBW, SGA infants, and preterm birth, have been reported in many previous studies;^5^^,^^9^^,^^10^^,^^12^^,^^13^ however, there was an overlap among the outcomes in these studies. LBW infants do not form a homogeneous group. For example, LBW babies who are SGA and those who show appropriate weight for gestational age (AGA) do not exhibit the same characteristics. To our knowledge, the relationship between maternal smoking and LBW has not been studied by classifying LBW infants into SGA and AGA and into preterm and full term. From the viewpoint of clinical and public health, to prevent LBW, it was important to obtain further information regarding maternal smoking during pregnancy as a risk factor for LBW.

Moreover, no population-based study has been conducted for identifying the difference in the etiologies of SGA and AGA babies and of preterm and full-term birth in LBW infants.

This study aimed to clarify the relationship of maternal smoking with LBW by classifying LBW infants into SGA and AGA babies and into preterm and full-term babies by using data from a prospective cohort study in Japan.

## METHODS

### Participants and Study Design

The study population comprised pregnant women who registered at the city office in Koshu City, Yamanashi Prefecture, Japan, between January 1, 1995, and December 31, 2000, and their children. The subjects were the participants of Project Koshu, a dynamic prospective cohort study consisting of pregnant women and their children in a Japanese rural area. This project commenced in 1988 and continues to date. Koshu City has a population of 27,000 with approximately 200 births occurring each year. We expected a high follow-up rate in this project because most of the people in this city had not migrated elsewhere. In the present study, we used a part of the data obtained from this project.

In Japan, pregnant women must register at a city office, and after delivery, the children must be registered by their parents. First, when the expectant mothers visited the city office for pregnancy registration, a questionnaire-based survey was conducted to ascertain their lifestyle habits, after obtaining informed consent. Over 95% of the expectant mothers in Koshu City registered before week 16 of pregnancy. Next, during their children’s medical checkup at a public health center, we obtained data regarding the gestational age at birth and birth weight that were recorded in the Maternal and Child Health Handbook by the obstetrician or midwife in charge of delivery.

In order to ensure confidentiality, the mothers and children were identified using unique numbers; these numbers were used to match the data obtained from the earlier pregnancy survey and those obtained at the children’s medical checkup performed when they reached 5 years of age.

We obtained informed consent from the participants of Project Koshu. This study was approved by the Ethical Review Board, Yamanashi University School of Medicine, based on the “Guidelines Concerning Epidemiological Research” (Ministry of Education, Culture, Sports, Science and Technology and Ministry of Health, Labour and Welfare) and was performed in cooperation with the Koshu City administration office.

### Exposure

The lifestyle immediately before pregnancy and during early pregnancy, including the smoking status during early pregnancy, was assessed based on a self-reported questionnaire administered at pregnancy registration. In this study, we used the following items as independent variables: sex of the child, birth order of the children, gestational age (weeks) at delivery, maternal age, maternal height, maternal body mass index (BMI) in the non-pregnant state, occupational status, smoking habits during early pregnancy, alcohol consumption during early pregnancy, breakfast habits, gestational age (weeks) at pregnancy registration, and maternal attitude toward pregnancy when the pregnancy was confirmed. Maternal body height and weight in the non-pregnant state were obtained from the data recorded in the Maternal and Child Health Handbook by the attending obstetrician or midwife. Maternal BMI was calculated according to World Health Organization standards (body weight (kg)/height (m^2^)).

### Outcome

Data regarding the sex of the infants, birth weight, birth height, and gestational age at delivery were obtained from the data recorded in the Maternal and Child Health Handbook by the obstetrician or midwife in charge of delivery. These data were based on birth registration. We used these data to diagnose the following outcomes: LBW, preterm birth, SGA, and AGA. LBW was defined as birth weight < 2,500 g, and preterm birth was defined as birth occurring at a gestational age < 37 weeks. SGA infants were diagnosed when the neonatal birth weight was below the 10th percentile of the standard birth weight curve for Japanese male and female infants.^14^ AGA infants were diagnosed when the neonatal birth weight was between the 10th and 90th percentiles of the standard birth weight curve. These standard birth weight curves were calculated for each sex and parity (order of delivery: 1^st^, 2^nd^, etc.).

### Statistical Analysis

First, to confirm whether the results of this cohort were consistent with previous results, we used multiple logistic regression analysis to clarify the risk factors of LBW, SGA infants, and preterm birth in this cohort. The dependent variables were birth weight (LBW or normal birth weight), intrauterine growth (SGA or not SGA), and gestational age at birth (preterm birth or no preterm birth). The independent variables were selected from previous reports regarding independent risk factors of LBW or SGA outcomes.

Next, we carried out 4 analyses using multiple logistic regression models to clarify the relationship of maternal smoking during pregnancy with SGA infants and with preterm LBW infants. We defined the following 4 types of cases in this cohort: (1) LBW SGA infants, (2) LBW AGA infants, (3) LBW preterm infants, and (4) LBW full-term infants. The independent variables were the same as those used in the cohort study regarding LBW, SGA, and preterm infants.

All analyses were conducted using SAS^®^ software, version 9.1 (SAS Institute Inc., Cary, North Carolina, USA).

## RESULTS

In this study, 1,329 pregnant women responded to the questionnaires administered at pregnancy registration. Of these, infant data were collected from 1,100 mothers (follow-up rate: 82.8%). Smoking during early pregnancy was reported by 72 (6.6%) mothers. Of the 81 (7.4%) LBW infants present, 47 (58.0%) were SGA, 34 (42.0%) were AGA, 25 (30.9%) were preterm, and 56 (69.1%) were full-term. The mean birth weight and gestational age (in weeks) of LBW, SGA, and preterm infants are listed in Table 1. The minimum birth weight recorded was 1,200 g, and the minimum gestational age was 31 weeks.

**Table 1.** Mean birth weight and gestational age (in weeks) of low birth weight (LBW), small for gestational age (SGA), and preterm infants.

Variable	LBW (n = 81)	SGA (n = 85)	Preterm (n = 40)
Birth weight (g)	2244.7 ± 275.3	2385.9 ± 318.3	2293.0 ± 460.1
Gestational age (weeks)	37.0 ± 1.9	38.7 ± 1.6	35.1 ± 1.2

mean ± standard deviation

The adjusted odds ratios (ORs) and 95% confidence intervals (CIs) for the maternal factors that influenced the LBW, SGA, and preterm birth outcomes are listed in Table 2. Maternal smoking habits during early pregnancy were associated with LBW (adjusted OR: 2.9; 95% CI: 1.2-6.9) and SGA (adjusted OR: 2.3; 95% CI: 1.1-5.1) outcomes. However, maternal smoking was not a risk factor for preterm birth (adjusted OR: 0.3; 95% CI: 0.04-2.3). An independent relationship was identified between female infants and preterm birth (adjusted OR: 0.4; 95% CI: 0.2-0.8). A reasonable relationship existed between the gestational age at birth and LBW (adjusted OR: 0.4; 95% CI: 0.3-0.4). Moreover, with regard to preterm birth, the maternal age at delivery was an independent risk factor (adjusted OR: 1.1; 95% CI: 1.02-1.2).

**Table 2.** Crude and adjusted odds ratios (ORs) and 95% confidence intervals (CIs) of maternal and fetal risk factors for the delivery of a low birth weight (LBW), small for gestational age (SGA), and preterm birth infant. The ORs and CIs were determined using logistic multivariable regression analysis.

Variables	LBW infants (n)	Non- LBW infants (n)	LBW				SGA infants (n)		Non- SGA infants (n)	SGA				Preterm infants (n)	Non- preterm infants (n)	Preterm birth			
		
			Crude		Adjusted^*†^					Crude		Adjusted^*‡^				Crude		Adjusted^*§^	
		
			OR	95% CI	OR	95% CI				OR	95% CI	OR	95% CI			OR	95% CI	OR	95% CI
Sex of infant																			
Male	41	535	1.0	(reference)	1.0	(reference)	41		535	1.0	(reference)	1.0	(reference)	26	550	1.0	(reference)	1.0	(reference)
Female	40	484	1.1	0.7-1.7	1.3	0.8-2.3	44		480	1.2	0.8-1.9	1.2	0.7-1.8	14	510	0.6	0.3-1.1	0.4	0.2-0.8

Birth order of the infant																			
Second or more	46	587	1.0	(reference)	1.0	(reference)	57		576	1.0	(reference)	1.0	(reference)	25	608	1.0	(reference)	1.0	(reference)
First	35	432	1.0	0.7-1.6	1.3	0.7-1.3	28		439	0.6	0.4-1.0	0.6	0.4-1.1	15	452	0.8	0.4-1.5	0.8	0.4-1.9
Gestational age at delivery (in weeks)					0.4	0.3-0.4													
Maternal age (in years)					1.0	0.9-1.04						1.0	0.97-1.1					1.1	1.02-1.2
Maternal body mass index before pregnancy (in kg/m^2^)					1.0	0.9-1.1						0.9	0.9-1.03					1.1	0.98-1.2

Maternal smoking during early pregnancy																			
Absent	72	956	1.0	(reference)	1.0	(reference)	76		952	1.0	(reference)	1.0	(reference)	39	989	1.0	(reference)	1.0	(reference)
Present	9	63	1.9	0.9-4.0	2.9	1.2-6.9	9		63	1.8	0.9-3.7	2.3	1.1-5.1	1	71	0.4	0.05-2.7	0.3	0.04-2.3

Maternal alcohol consumption during early pregnancy																			
Absent	73	912	1.0	(reference)	1.0	(reference)	76		909	1.0	(reference)	1.0	(reference)	36	949	1.0	(reference)	1.0	(reference)
Present	7	97	0.9	0.4-2.0	1.1	0.4-2.8	8		96	1.0	0.5-2.1	0.9	0.4-2.0	4	100	1.1	0.4-3.0	0.6	0.1-2.5

Maternal breakfast consumption																			
“I don’t skip breakfast”	62	769	1.0	(reference)	1.0	(reference)	69		762	1.0	(reference)	1.0	(reference)	30	801	1.0	(reference)	1.0	(reference)
“I occasionally skip breakfast”	19	250	0.9	0.6-1.6	0.7	0.4-1.4	16		253	0.7	0.4-1.2	0.8	0.4-1.4	10	259	1.0	0.5-2.1	1.5	0.6-3.4

Maternal occupational status																			
None	43	543	1.0	(reference)	1.0	(reference)	47		539	1.0	(reference)	1.0	(reference)	21	565	1.0	(reference)	1.0	(reference)
Working	38	476	1.0	0.6-1.6	1.2	0.7-2.1	38		476	1.0	0.6-1.6	1.1	0.7-1.7	19	495	1.0	0.5-1.9	1.3	0.6-2.6

Maternal attitude toward pregnancy when pregnancy was confirmed																			
Positive attitude	71	914	1.0	(reference)	1.0	(reference)	75	910		1.0	(reference)	1.0	(reference)	35	950	1.0	(reference)	1.0	(reference)
Negative attitude	10	105	0.9	0.4-2.1	0.9	0.4-2.1	10	105		1.2	0.6-2.3	1.0	0.5-2.0	5	110	1.2	0.5-3.2	1.5	0.5-4.0

Time of registration of pregnancy																			
Early (<12 gestational weeks)	53	724	1.0	(reference)	1.0	(reference)	60	717		1.0	(reference)	1.0	(reference)	25	752	1.0	(reference)	1.0	(reference)
Late (≥12 gestational weeks)	28	295	1.3	0.8-2.1	1.2	0.7-2.1	25	298		1.0	0.6-1.6	1.0	0.6-1.7	15	308	1.5	0.8-2.8	1.7	0.8-3.4

* : Adjusted for all presented variables

† : LBW (74) and non-LBW (957) infants for whom responses to all questionnaires in this model were available were analyzed.

‡ : SGA (81) and non-SGA (950) infants for whom responses to all questionnaires in this model were available were analyzed.

§ : Preterm (35) and full-term (996) infants for whom responses to all questionnaires in this model were available were analyzed.

Next, we carried out 2 multivariable analyses to compare the risk factors between LBW SGA infants and LBW AGA infants. Maternal smoking during early pregnancy was identified as a risk factor for LBW and SGA outcomes (adjusted OR: 3.8; 95% CI: 1.6-9.1). On the other hand, no risk factor for the latter outcome in this analysis. (Table 3)

**Table 3.** Crude and adjusted odds ratios (ORs) and 95% confidence intervals (CIs) of maternal and fetal risk factors for the delivery of a low birth weight (LBW) small for gestational age (SGA) or a LBW appropriate for gestational age (AGA) infant. The ORs and CIs were determined using logistic multivariable regression analysis.

Variables	LBW SGA infants (n)	Non- LBW SGA infants (n)	LBW SGA infants				LBW AGA infants (n)	Non- LBW AGA infants (n)	LBW AGA infants			
	
			Crude		Adjusted^*†^				Crude		Adjusted^*‡^	
	
			OR	95% CI	OR	95% CI			OR	95% CI	OR	95% CI
Sex of infant												
Male	26	550	1.0	(reference)	1.0	(reference)	15	561	1.0	(reference)	1.0	(reference)
Female	21	503	0.9	0.5-1.6	0.9	0.5-1.7	19	505	1.4	0.7-2.8	1.0	0.5-2.1

Birth order of the infant												
Second or more	29	604	1.0	(reference)	1.0	(reference)	17	616	1.0	(reference)	1.0	(reference)
First	18	449	0.8	0.5-1.5	0.9	0.4-1.7	17	450	1.4	0.7-2.7	1.3	0.5-2.9

Maternal smoking during early pregnancy												
Absent	39	989	1.0	(reference)	1.0	(reference)	33	995	1.0	(reference)	1.0	(reference)
Present	8	64	3.2	1.4-7.1	3.8	1.6-9.1	1	71	0.4	0.1-3.2	0.4	0.1-3.0

Maternal alcohol consumption during early pregnancy												
Absent	43	942	1.0	(reference)	1.0	(reference)	30	955	1.0	(reference)	1.0	(reference)
Present	4	100	0.9	0.3-2.5	0.9	0.3-2.5	3	101	0.9	0.3-3.2	0.7	0.2-3.2

Maternal breakfast consumption												
“I don’t skip breakfast”	38	793	1.0	(reference)	1.0	(reference)	24	807	1.0	(reference)	1.0	(reference)
“I occasionally skip breakfast”	9	260	0.7	0.3-1.5	0.6	0.3-1.5	10	259	1.3	0.6-2.8	1.3	0.6-3.1

Maternal occupational status												
None	28	558	1.0	(reference)	1.0	(reference)	15	571	1.0	(reference)	1.0	(reference)
Working	19	495	0.8	0.4-1.4	0.8	0.4-1.5	19	495	1.5	0.7-2.9	2.0	0.9-4.6

Maternal attitude toward pregnancy when pregnancy was confirmed												
Posithive attitude	41	944	1.0	(reference)	1.0	(reference)	30	955	1.0	(reference)	1.0	(reference)
Negative attitude	6	109	1.3	0.5-3.1	0.9	0.3-2.3	4	111	1.1	0.4-3.3	1.6	0.5-4.9

Time of registration of pregnancy												
Early (<12 gestational weeks)	31	746	1.0	(reference)	1.0	(reference)	22	755	1.0	(reference)	1.0	(reference)
Late (≥12 gestational weeks)	16	307	1.3	0.7-2.3	1.2	0.6-2.3	12	311	1.3	0.6-2.8	1.4	0.7-3.1

* : Adjusted for all presented variables, maternal age at delivery, and maternal body mass index before pregnancy.

† : LBW SGA (46) and non-LBW SGA (985) infants for whom responses to all questionnaires in this model were available were analyzed.

‡ : LBW AGA (28) and non-LBW AGA (1003) infants for whom responses to all questionnaires in this model were available were analyzed.

In addition, we compared the risk factors between LBW preterm infants and LBW full-term infants. In the former, late registration of pregnancy was the only risk factor identified (adjusted OR: 2.9; 95% CI: 1.2-7.0), and no other risk factors, including maternal smoking (adjusted OR: 0.5; 95% CI: 0.1-3.7), were identified. In the latter, maternal smoking during early pregnancy was identified as a risk factor (adjusted OR: 3.1; 95% CI: 1.3-7.2). (Table 4)

**Table 4.** Crude and adjusted odds ratios (ORs) and 95% confidence intervals (CIs) of maternal and fetal risk factors for the delivery of a low birth weight (LBW) preterm or a LBW full-term infant. The ORs and CIs were determined using logistic multivariable regression analysis.

Variables	LBW preterm infants (n)	Non- LBW preterm infants (n)	LBW preterm infants				LBW full-term infants (n)	Non- LBW full-term infants (n)	LBW full-term infants			
	
			Crude		Adjusted^*†^				Crude		Adjusted^*‡^	
	
			OR	95% CI	OR	95% CI			OR	95% CI	OR	95% CI
Sex of infant												
Male	15	561	1.0	(reference)	1.0	(reference)	26	550	1.0	(reference)	1.0	(reference)
Female	10	514	0.7	0.3-1.6	0.4	0.2-.1	29	495	1.2	0.7-2.1	1.2	0.7-2.2

Birth order of the infant												
Second or more	14	619	1.0	(reference)	1.0	(reference)	32	601	1.0	(reference)	1.0	(reference)
First	11	456	1.1	0.5-2.4	1.1	0.4-3.0	23	444	1.0	0.6-1.7	0.9	0.5-1.7

Maternal smoking during early pregnancy												
Absent	24	1004	1.0	(reference)	1.0	(reference)	47	981	1.0	(reference)	1.0	(reference)
Present	1	71	0.6	0.1-4.4	0.5	0.1-3.7	8	64	2.6	1.2-5.8	3.1	1.3-7.2

Maternal alcohol consumption during early pregnancy												
Absent	22	963	1.0	(reference)	1.0	(reference)	51	934	1.0	(reference)	1.0	(reference)
Present	3	101	1.3	0.4-4.4	1.2	0.3-5.6	3	101	0.5	0.2-1.8	0.5	0.2-1.7

Maternal breakfast consumption												
“I don’t skip breakfast”	19	812	1.0	(reference)	1.0	(reference)	42	789	1.0	(reference)	1.0	(reference)
“I occasionally skip breakfast”	6	263	1.0	0.4-2.5	1.1	0.4-3.2	13	256	1.0	0.5-1.8	0.9	0.4-1.7

Maternal occupational status												
None	11	575	1.0	(reference)	1.0	(reference)	32	554	1.0	(reference)	1.0	(reference)
Working	14	500	1.5	0.7-3.3	1.9	0.8-4.8	23	491	0.8	0.5-1.4	1.0	0.5-1.7

Maternal attitude toward pregnancy when pregnancy was confirmed												
Positive attitude	23	962	1.0	(reference)	1.0	(reference)	47	938	1.0	(reference)	1.0	(reference)
Negative attitude	2	113	0.7	0.2-3.2	0.8	0.2-3.8	8	107	1.5	0.7-3.2	1.3	0.6-3.0

Time of registration of pregnancy												
Early (<12 gestational weeks)	13	764	1.0	(reference)	1.0	(reference)	39	738	1.0	(reference)	1.0	(reference)
Late (≥12 gestational weeks)	12	311	2.3	1.0-5.0	2.9	1.2-7.0	16	307	1.0	0.5-1.8	1.0	0.4-1.7

* : Adjusted for all presented variables, maternal age at delivery, and maternal body mass index before pregnancy.

† : LBW preterm (21) and non-LBW preterm (1010) infants for whom responses to all questionnaires in this model were available were analyzed.

‡ : LBW full-term (52) and non-LBW full-term (979) infants for whom responses to all questionnaires in this model were available were analyzed.

## DISCUSSION

It is estimated that 40% of all cases of LBW occur due to hereditary factors, and the remaining 60% occur due to environmental factors.^15^ Maternal smoking during early pregnancy is a major risk factor for LBW; however, other risk factors for LBW remain unknown. Moreover, because LBW infants do not form a homogeneous group, we supposed that there are multiple etiologies responsible for LBW. In order to clarify these issues, we carried out an epidemiologic study by using a prospective cohort of pregnant women in a Japanese rural area.

The follow-up rate in this study was 82.8%; the most common reasons for discontinuing follow up might be migration to another area or miscarriage. Moreover, the participants in our study included 3 infants with very LBW. Although we could not obtain information regarding the complication(s) in these 3 cases, the mothers of these infants did not smoke during early pregnancy. Thus, our results pertaining to the association between maternal smoking and outcome of pregnancy might be an underestimation.

First, we carried out a cohort study to clarify the relationship between maternal smoking during early pregnancy and pregnancy outcomes, such as LBW, in this population. Our results suggested that maternal smoking during pregnancy was a risk factor for LBW and SGA outcomes. These results were similar to those of previous studies.^5^^,^^9^^,^^10^^,^^12^^,^^13^ However, regarding the association between maternal smoking during pregnancy and preterm birth, contradictory results have been reported in previous studies. Some studies indicated the absence of such an association,^16^^,^^17^ whereas others indicated that maternal smoking during pregnancy was a risk factor for preterm birth.^18^^,^^19^ Even if an actual relationship exists between maternal smoking and preterm birth, our result might be attributed to the small sample size because the effect of smoking during pregnancy on preterm birth was suggested to be smaller than that on LBW and SGA.^18^ Moreover, maternal age at delivery and the sex of the infants were associated with preterm birth. These results were consistent with those of previous studies.^19^^-^^22^

Next, we carried out 4 analyses to specify the risk factors for LBW SGA, LBW AGA, LBW preterm, and LBW full-term infants in this cohort. This cohort might be considered similar to the general population, based on the results of a previous cohort study regarding LBW, SGA infants, and preterm birth. Therefore, the bias influencing each group, such as socioeconomic background, might have been minimized in this study. In these analyses, we clarified that maternal smoking during early pregnancy was a major risk factor for LBW with SGA outcome but not for LBW with AGA outcome. These results in a prospective population-based study showed that there was a difference in the etiology of LBW infants. LBW AGA or LBW preterm outcomes were associated with other risk factors that were not considered in this study, such as periodontal disease or bacterial vaginosis.^23^^-^^29^ However, no consistent conclusion was reached, especially, regarding an association between periodontal disease and LBW preterm infants.^30^ Further studies are required to reveal these risk factors.

Moreover, the effect of maternal smoking during early pregnancy as a risk factor for LBW preterm infants was similar to its effect as a risk factor for LBW AGA infants, and its effects as a risk factor for LBW full-term and LBW SGA infants were similar. In this study, LBW preterm infants accounted for approximately 70% LBW AGA infants, while LBW full-term infants constituted approximately 80% LBW SGA infants. Our results reflect a strong association between intrauterine growth and gestational age at delivery in LBW infants.

Our results also indicated that late pregnancy registration (≥12 weeks) was a risk factor for LBW preterm infants. A previous study has reported that women with unwanted pregnancies had an increased likelihood of preterm delivery.^31^ Late registration of pregnancy might be due to unawareness of or unwanted pregnancy. Therefore, our result was consistent with that of the previous report.

Nevertheless, this study had some limitations. First, we recruited the participants over a 6-year period. During this time, some changes occurred in the participants’ background, such as increase in the knowledge of perinatal risk factors. These effects were beyond our control. Moreover, it is possible that women who delivered 2 or more children during this period might have participated 2 or more times in this study. However, we collected data from the pregnant women at the time pregnancy registration, and the BMI of mothers, which were the main genetic factors of physical development of the fetus, were controlled in statistical analysis. Therefore, we thought that the effect of this limitation of our study might be minimized.

Second, we could not collect data regarding clinical complications such as periodontal disease or bacterial vaginosis because our study was based on a public health activity. Further studies to reveal these clinical risk factors and the interaction between these factors and previously well-known risk factors are required.

Third, we could not obtain data regarding maternal smoking trends at various stages of pregnancy.

Fourth, it is possible that the data regarding the analysis of preterm infants was insufficient because of the relatively small number of cases included. However, the OR of maternal smoking during pregnancy being associated with the preterm delivery was lesser than that of it being associated with LBW SGA infants. This result suggested the existence of different risk factors for LBW SGA infants and preterm birth.

Despite these limitations, our study was based on community-based prospective data and had a high follow-up rate. Thus, our results were verified to have high internal validity. Moreover, the results of our cohort study were consistent with those of previous studies. This indicated that our questionnaire correctly reflected the lifestyles of the participants. Therefore, our results were verified to have high external validity as well.

This prospective study indicated a difference in the risk factors for LBW SGA infants and LBW preterm infants. Moreover, LBW and preterm birth might be associated with other risk factors that were not considered in this study, such as periodontal disease or bacterial vaginosis.^23^^-^^29^ These results suggested that for the prevention of LBW, not only abstinence from smoking during pregnancy but also other methods such as establishing a clinical setting should be adopted.
